# Gender differences in body composition, dietary patterns, and physical activity: insights from a cross-sectional study

**DOI:** 10.3389/fnut.2024.1414217

**Published:** 2024-07-11

**Authors:** Mauro Lombardo, Alessandra Feraco, Andrea Armani, Elisabetta Camajani, Stefania Gorini, Rocky Strollo, Elvira Padua, Massimiliano Caprio, Alfonso Bellia

**Affiliations:** ^1^Department for the Promotion of Human Science and Quality of Life, San Raffaele Open University, Rome, Italy; ^2^Laboratory of Cardiovascular Endocrinology, San Raffaele Research Institute, IRCCS San Raffaele Roma, Rome, Italy; ^3^Department of Systems Medicine, University of Rome “Tor Vergata”, Rome, Italy

**Keywords:** gender, body composition, eating behaviour, physical activity patterns, cross-sectional study

## Abstract

**Introduction:**

This study investigates the interplay between body composition, dietary patterns, and physical activity across genders, focusing on gender-specific differences in food preferences and eating behaviors. Understanding these interactions is crucial for developing targeted nutritional and lifestyle interventions.

**Methods:**

A cross-sectional study was conducted with 1,333 participants (58.7% female, 41.3% male), aged 18–65 years. Participants were categorized into tertiles based on their fat mass to fat-free mass (FM-to-FFM) ratio. Data on dietary choices, eating behaviors, and physical activity were collected and analyzed to identify gender-specific trends.

**Results:**

Significant gender-specific differences were observed in food preferences and eating behaviors. Males experienced greater hunger in the late afternoon, while females felt more hunger in the morning. Males showed a preference for processed and red meats, whereas females preferred cooked vegetables. Eating behaviors such as meal skipping, uncontrolled eating, nocturnal eating, and taste preferences (sweet or salty) varied distinctly between FM-to-FFM tertiles and genders. Higher FM-to-FFM ratios correlated with lower physical activity levels, particularly in strength training and general sports engagement.

**Discussion:**

These findings highlight the complex interactions between body composition, dietary habits, and lifestyle factors, emphasizing gender-specific differences. The results suggest that body composition and BMI significantly impact health-related behaviors, necessitating tailored interventions to address these differences and promote healthier lifestyles.

## 1 Introduction

In recent decades, research has highlighted significant gender differences in hormonal pathways, medical parameters, food preferences, and eating behaviour, emphasising the need for personalised nutritional strategies (1, 2). These differences, rooted in evolutionary, biological and socio-cultural factors (3), underline the importance of developing personalised nutritional strategies that take into account the specific needs and preferences of men and women (4). In fact, women tend to show a greater inclination toward healthier food choices (5), whereas men favour high-calorie foods, often for the pleasure of consumption (6).

The importance of gender differences in nutrition extends beyond simple preference or aversion to certain foods; these distinctions reflect complex interactions between genetic, environmental and psychosocial factors (7). Observed trends in men’s and women’s food consumption are not only the result of individual choices, but are influenced by a range of cultural, social and psychological aspects that shape food perceptions and practices (8). These influences need to be carefully considered in order to fully understand the role of gender in dietary decisions and prevention strategies for diet-related diseases (9).

In this study, we aim to explore how differences in emotional and psychological reactions to food may influence the susceptibility of men and women to develop obesity and eating disorders. Moreover, our analysis aims to provide insights into the prevention of cardiometabolic diseases by analysing the contribution of gender and body composition differences in eating habits, food preferences, and nutritional knowledge, promoting a more holistic approach to nutrition (10). Previous research has shown how gender can influence not only body fat distribution and muscle composition, but also how these physical characteristics can in turn modulate food choices and tastes (11). Men and women show significant differences in body composition that, together with divergences in reward circuits and neural responses to food stimuli, could contribute to distinct food consumption patterns (12). Examining these relationships could reveal specific gender-related patterns of vulnerability and resilience, offering crucial insights into the design of more effective and personalised nutritional interventions (13). Understanding how body composition interacts with gendered food preferences is critical for addressing challenges related to obesity, eating disorders and cardiometabolic diseases, promoting preventive and therapeutic strategies that reflect the diversity of human experiences.

## 2 Methods

### 2.1 Recruitment of participants

In this cross-sectional study, we examined a group of adult and adolescent individuals attending a medical centre specialising in nutrition and metabolism located in Rome, Italy. Participant recruitment began in May 2023 and continued until November 2023, during which time we collected data from 1,500 surveys and conducted medical examinations. Participants younger than 18 or older than 65, or those with a BMI below 18.5 kg/m^2^ or above 40 kg/m^2^, were excluded from the study to ensure a more homogeneous sample.

Subjects with psychiatric disorders, pregnancy or breastfeeding, alcohol addiction were excluded. Following these exclusion criteria, 167 participants were excluded from the analysis. The ethical procedures of the study, including the consent form, were approved by the IRCCS San Raffaele Ethics Committee (registration number RP 23/13), ensuring adherence to the ethical guidelines outlined in the Declaration of Helsinki and its subsequent updates.

### 2.2 Body composition evaluation

All participants underwent a thorough medical evaluation that included a dietary history, physical examination and body composition (BC) assessment. Body measurements were performed after an overnight fast, with participants wearing only their undergarments. The evaluation process included a comprehensive examination of the participants’ eating habits, physical health status and BC. Calibrated electronic scales capable of supporting up to 250 kg were used for the weighing process. These scales were placed on a flat, hard, non-carpeted surface. The participants, dressed in light clothing and barefoot, stood still on the scales while a clinical assistant recorded their weight to the nearest gram. Two weight readings were taken to verify accuracy and a third if the first two differed by more than 100 grams; the two closest readings were selected for analysis. Height was measured with a stadiometer placed on a flat, hard surface. Participants stood barefoot, without hairstyles that could influence their height and facing the clinic assistant with their heads aligned with the Frankfurt floor. They were asked to stand with their backs and heels gently touching the stadiometer, arms at their sides, legs straight, knees together and feet flat. Two height measurements were recorded, and a third was taken if there was a discrepancy of more than 0.1 cm between them; the two closest measurements were used for further analysis. Body composition, including percentages and kilograms of fat mass (FM), fat-free mass (FFM), and total body water (TBW), was determined using the Tanita BC-420 MA bioimpedance metre, validated for accuracy against the BodPod. (14), allows measurements to be taken in a standing position without the need for electrodes and provides an accuracy of up to 100 grams. The pre-assessment guidelines required participants to have been fasting for at least 3 h since their last meal or drink, to have spent at least 12 h from strenuous physical activity, and to avoid excess food, drink and alcohol in the 12 h prior to measurement. In addition, women were recommended to avoid measurement during their menstrual cycle.

### 2.3 Online questionnaire

Before the medical examination, the participants completed an online questionnaire concerning their eating habits. Afterwards, they discussed the results with the medical staff. The questionnaire was developed based on previous validated food preference surveys, with modifications adapted to the study population. A pilot test was conducted with a small subset of participants to ensure clarity and relevance, with minor modifications to the wording of some questions. The questionnaire, which was accessible via a link allowing participants to choose whether or not to participate in the study, was self-administered and structured to be completed in approximately 30 min from any device with an Internet connection. Although no formal validation was conducted, the survey was comparable to previously validated food preference questionnaires (15). The anonymously recorded answers were divided into four sections, consistent with previous food preference questionnaires (16, 17). The first section investigated the frequency and type of meals, times of increased appetite, habits such as skipping meals or eating fast, and the quality of sleep, including any disturbances. The second part of the survey focused on food preferences for a variety of foods, such as dairy products, meat and its alternatives, vegetables, fruit, cereals and dark chocolate. Next, consumption habits of meals, water, alcohol and sugary drinks were explored, as well as recollection of food intake in the last 24 h. The last part assessed the presence of physical activity, the number of hours devoted to sport per week and the type of activity practised.

### 2.4 Statistical analysis

In the course of this study, we explored the food preferences of a representative sample, dividing the responses according to BMI categories, specifically: Normal Weight (18.5–24.9), Overweight (25.0–29.9), First Degree Obesity (30–34.9) and Second Degree Obesity (35+). Analysing the frequency of consumption of 18 different types of food, we calculated the percentages of affirmative responses for each food. To assess the existence of statistically significant differences in food preferences between the BMI categories, the chi-square test was applied. To examine the relationship between body composition and food preferences, we divided the participants into categories based on the ratio of FM to lean mass FFM, organising the study population into tertiles according to the distribution of the FM/FFM ratio. Chi-square tests were chosen for the analysis of categorical data due to their robustness in assessing the distribution of sports participation across categories. To account for multiple comparisons, a Bonferroni correction was applied to control for Type I error. This classification made it possible to identify three distinct groups: Low, Medium and High, based on their FM/FFM ratio values. The analysis of the results was conducted using ANOVA and Kruskal-Wallis tests, depending on the nature of the variables, to determine the statistical significance of the observed differences. We investigated eating habits, including daily hunger frequency, propensity to skip meals and speed of consumption, through the use of the chi-square test of independence to assess differences between BMI categories. Taste preference was assessed via a self-reported scale from 1 (preference for sweet) to 10 (preference for salty), with analysis of variance (ANOVA) to explore differences in preference between tertiles and between genders. Finally, we analysed sports participation patterns across different body composition tertiles based on the FM/FFM ratio, segmented by gender. The data were collected through a detailed questionnaire that categorised each participant’s primary sport into five categories: no sport, endurance sport, strength training, team sport, and skill sport. Chi-square tests were employed to examine the distribution of sports participation within these categories and to evaluate the statistical significance of observed participation patterns.

## 3 Results

In this study, we examined the differences in demographic and clinical characteristics between the patient groups according to BMI categories. Our sample consisted of 1,333 patients (age 18–65), with a gender distribution of 58.7% female and 41.3% male (Table 1). The analysis showed significant differences between the groups across all considered variables, such as age, weight, BMI, FM, abdominal circumference, FFM, body water, FM-to-FFM ratio, body protein, and basal metabolic rate.

**Table 1:** Demographic and clinical characteristics of patients by BMI category.

BMI							
		Total (*n*)	18.5–24.9	25.0–29.9	30–34.9	>35	*p*-value
Number of patients		1,333	391	531	275	135	<0.0001
		F: 782 (58.7%), M: 551 (41.3%)	F: 260 (66.5%), M: 131 (33.5%)	F: 306 (57.6%), M: 225 (42.4%)	F: 142 (51.6%), M: 133 (48.4%)	F: 73 (54.1%), M: 62 (45.9%)	<0.0001
Age	yrs	39.4 ± 11.7	35.3 ± 10.5	40.1 ± 11.8	41.3 ± 11.8	44.8 ± 11.5	<0.0001
Weight	kg	80.1 ± 17.5	64.7 ± 8.4	77.8 ± 10.1	92.3 ± 11.4	109.4 ± 16.5	<0.0001
BMI	Kg/m^2^	28.2 ± 5.1	22.9 ± 1.5	27.3 ± 1.5	32.2 ± 1.4	38.6 ± 3.5	<0.0001
FM	kg	25.1 ± 10.6	15.3 ± 4.8	23.3 ± 5.2	32.8 ± 5.8	44.8 ± 8.9	<0.0001
FM	%	30.7 ± 8.9	23.9 ± 7.2	30.3 ± 7.0	35.9 ± 6.7	41.2 ± 6.8	<0.0001
AC	cm	97.0 ± 13.9	83.3 ± 7.4	95.6 ± 7.6	107.8 ± 6.8	119.8 ± 11.0	<0.0001
FFM	kg	52.3 ± 11.2	46.8 ± 8.4	51.7 ± 10.2	56.5 ± 10.9	61.3 ± 13.2	<0.0001
Body water	kg	38.9 ± 8.4	34.3 ± 6.1	38.2 ± 7.1	42.5 ± 8.0	47.2 ± 10.2	<0.0001
FM-to-FFM	Ratio	0.5 ± 0.2	0.3 ± 0.1	0.5 ± 0.2	0.6 ± 0.2	0.8 ± 0.2	<0.0001
Body protein	Kg	13.4 ± 3.1	12.5 ± 2.5	13.6 ± 3.2	14.0 ± 3.2	14.1 ± 3.6	<0.0001
BMR	Kcal	1,659 ± 342	1,477 ± 248	1,635 ± 297	1805 ± 325	1984 ± 409	<0.0001
<€20.000	N (%)	205 (15.4%)	65 (16.6%)	89 (16.8%)	41 (14.9%)	9 (6.7%)	<0.0001
€20.000–€40.000		921 (69.1%)	271 (69.3%)	347 (65.3%)	197 (71.6%)	106 (78.5%)	
€40.000–€60.000		170 (12.8%)	40 (10.2%)	79 (14.9%)	33 (12.0%)	18 (13.3%)	
>€60.000		37 (2.8%)	15 (3.8%)	16 (3.0%)	4 (1.5%)	2 (1.5%)	

Presents a comparison of the demographic and clinical characteristics of the patients, subdivided according to Body Mass Index (BMI) categories: 18.5–24.9 (normal weight), 25.0–29.9 (overweight), 30–34.9 (first degree obesity), and > 35 (second degree obesity or greater). Total number of patients (*n*), gender distribution (F: females, M: males), age, weight, BMI, fat mass (FM kg and FM %), abdominal circumference (AC), lean body mass (FFM), body water, fat-to-fat mass ratio (FM-to-FFM), body protein, basal metabolic rate (BMR) and income distribution were included. The *p*-values were calculated using the Analysis of Variance (ANOVA) for continuous variables with normal distribution and the Kruskal-Wallis test for non-normal or categorical variables in order to assess statistically significant differences between the groups.

**Figure 1:**
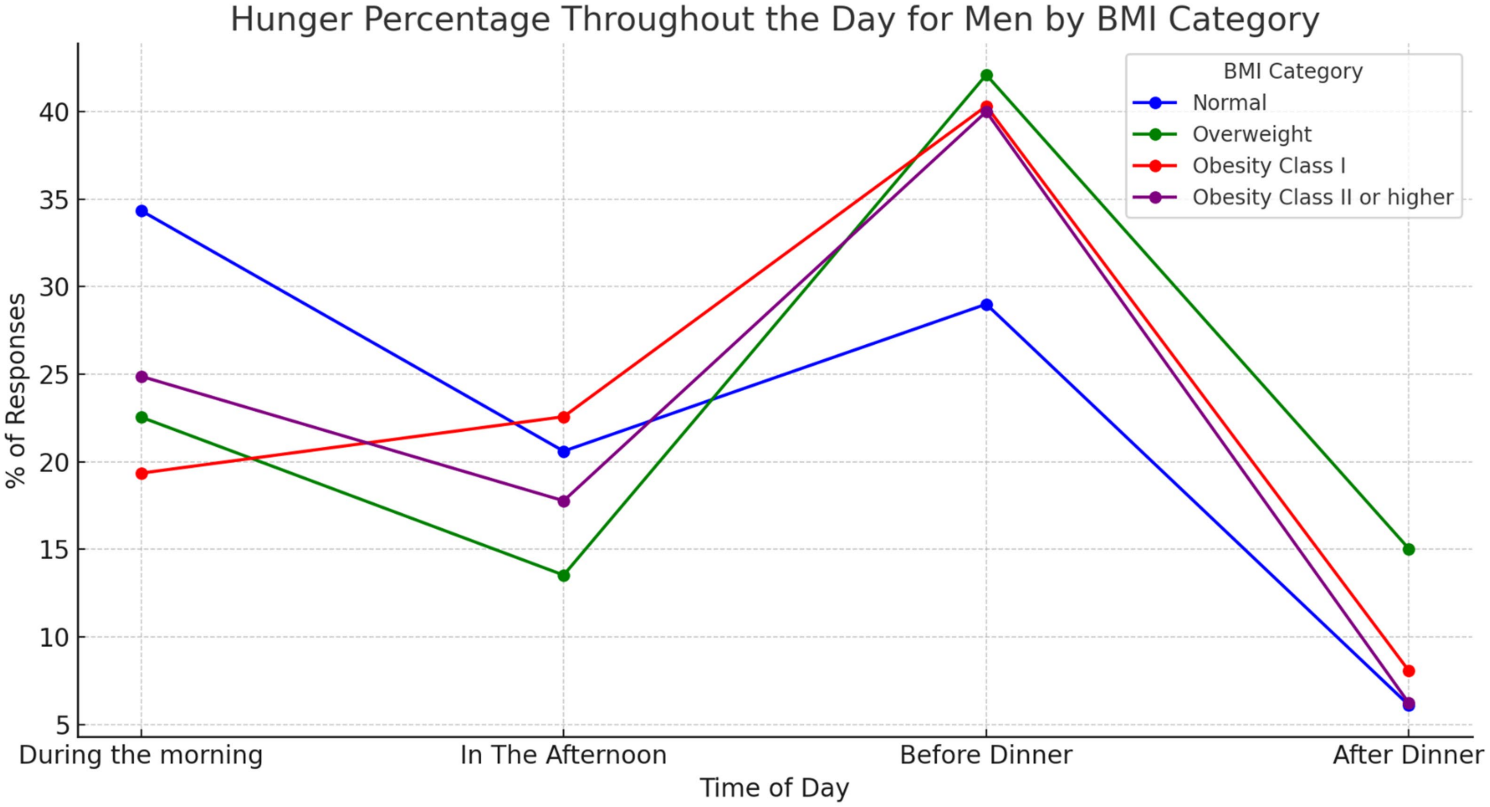
Answers to the question “when am I hungriest during the day” for males, ranked by BMI category. Illustrates how men’s perception of hunger fluctuates throughout the day, categorised by Body Mass Index (BMI). Responses indicating “I am always hungry” are excluded. Our analysis revealed statistically significant differences (*p* = 0.0022) in how hungry men feel at various times of the day. Additionally, hunger perception exhibits significant variation between sexes (*p* = 0.00074).

**Figure 2:**
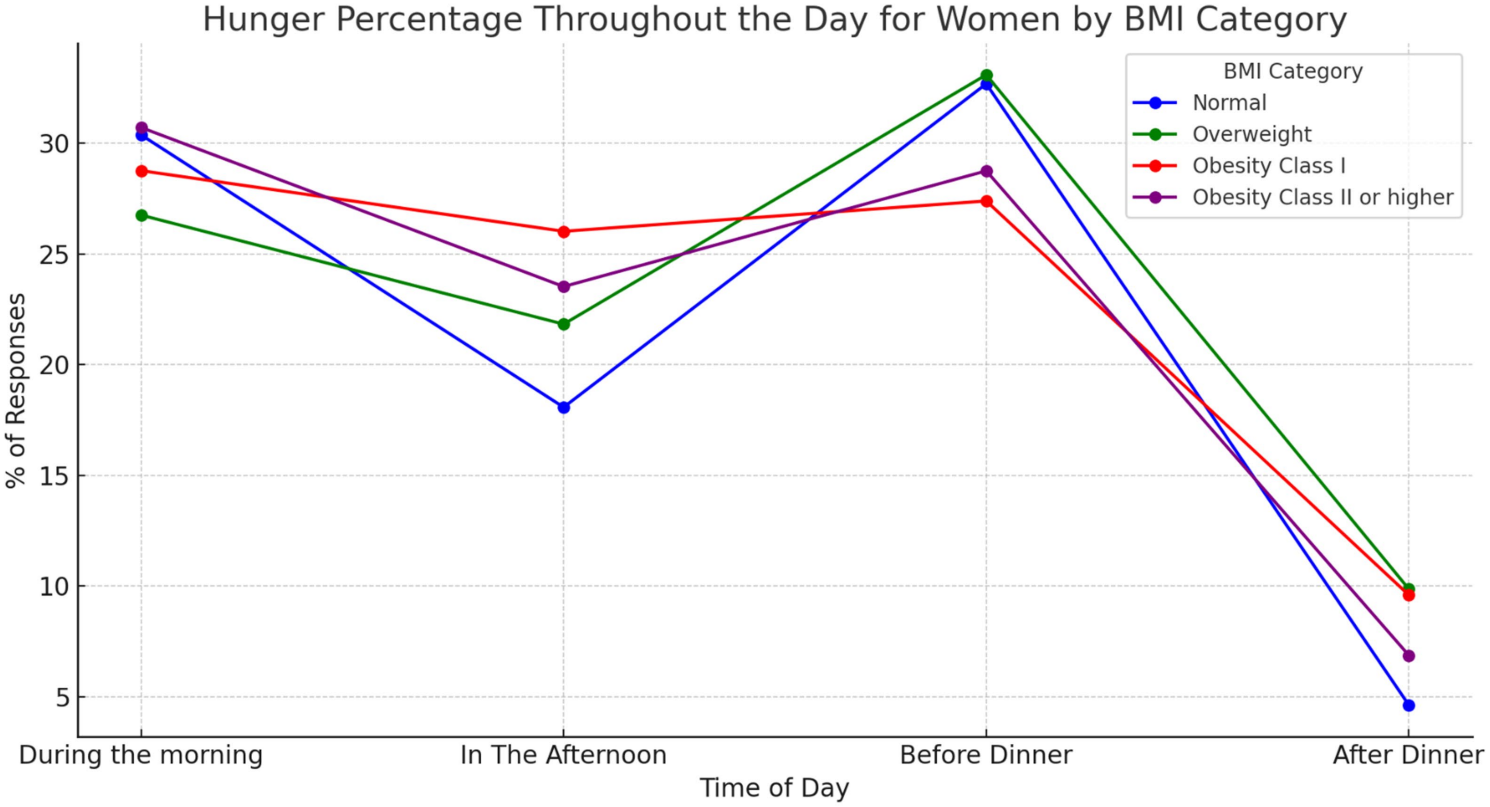
Answers to the question “when am I hungriest during the day” for females, ranked by BMI category. Illustrates how women’s perception of hunger fluctuates throughout the day, categorised by Body Mass Index (BMI). Responses indicating “I am always hungry” are excluded. Our analysis revealed statistically significant differences (*p* = 0.0022) in how hungry women feel at various times of the day. Additionally, hunger perception exhibits significant variation between sexes (*p* = 0.00074).

We found statistically significant differences in responses related to when individuals feel hungry during the day (*p* = 0.0022). These differences are significant when comparing the sexes (*p* = 0.00074). Males tend to report a greater perception of hunger in the late afternoon and before dinner, especially in the ‘overweight’ and ‘obesity’ categories (Figure 1). Females show a greater perception of hunger during the morning and greater homogeneity between the different BMI ranges in their responses (Figure 2).

**Figure 3:**
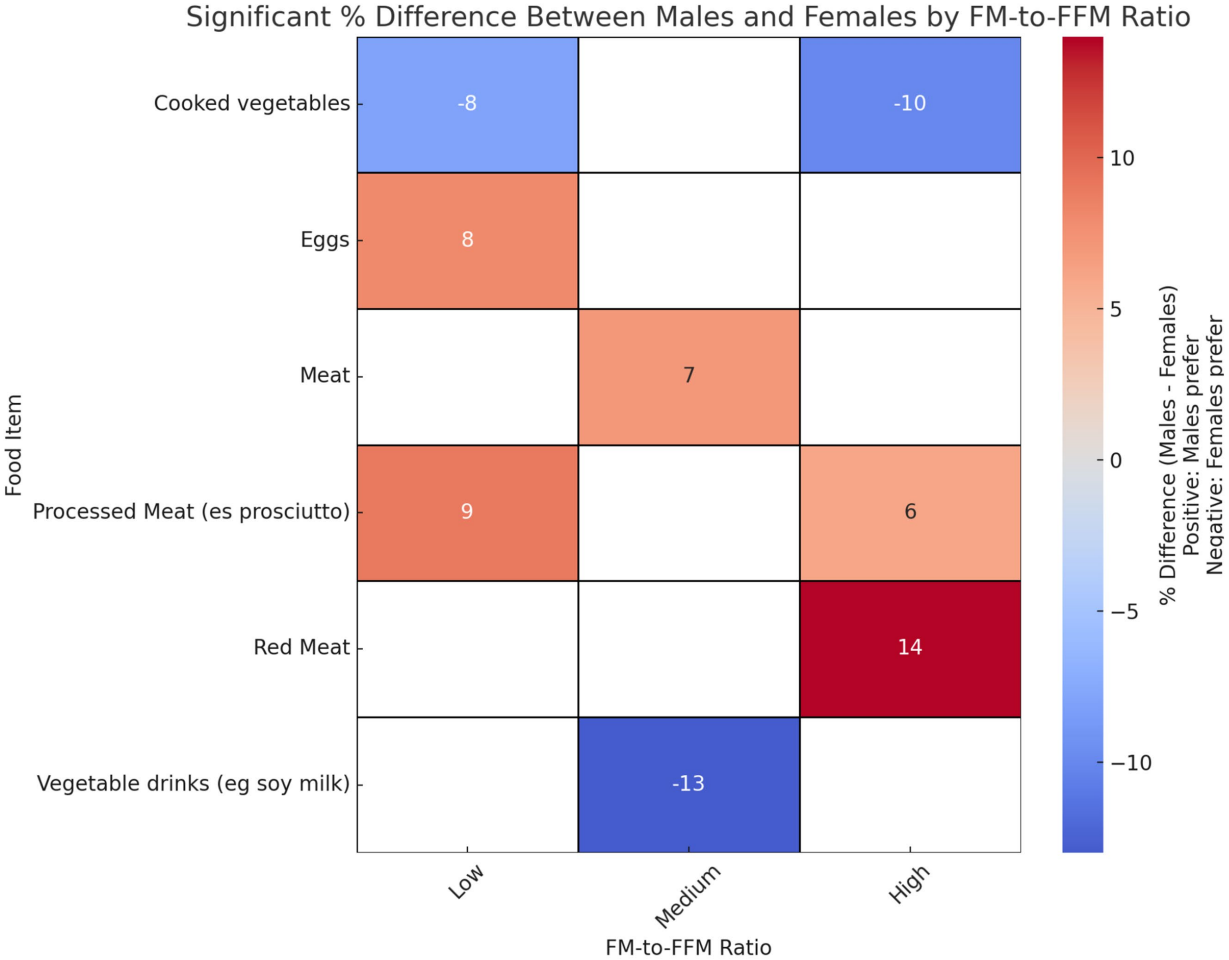
Gender differences in food preferences: an analysis of FM/FFM tertiles. Heatmap showing the significant percentage differences between males and females in dietary preferences across tertiles of FM-to-FFM ratio. Positive values indicate a higher preference among males, and negative values indicate a higher preference among females. *p*-values indicating significance of differences are as follows: Processed Meat (e.g., prosciutto): *p* = 0.0287, Eggs: *p* = 0.0352, Cooked Vegetables: *p* = 0.0092 for tertile 1. Vegetable Drinks (e.g., soy milk): *p* = 0.0252, Meat: *p* = 0.0441 for tertile 2. Red Meat: *p* = 0.0132, Processed Meat (e.g., prosciutto): *p* = 0.0360, Cooked Vegetables: *p* = 0.0002 for tertile 3.

The analysis revealed gender differences in food preferences between body composition groups, defined by tertiles of the FM-to-FFM ratio (Figure 3 and Supplementary Figure S1). In the lowest tertile, males showed a preference of 90.2% for Processed Meat (e.g., ham), compared to 81.5% for females (*p* = 0.029). For Eggs, males recorded 91.8%, compared to 83.5% for females (*p* = 0.035). Regarding Cooked Vegetables, males scored 87.5%, compared to 95.4% for females (*p* = 0.009). In the medium FM-to-FFM tertile, females showed 28.2% preference for Vegetable Beverages (e.g., soya milk), compared to 40.7% for males (*p* = 0.025). Finally, in the highest tertile, males recorded 92.3% preference for Red Meat, compared to 78.5% for females (*p* = 0.013). For Processed Meat, males scored 95.1%, compared to 88.9% for females (*p* = 0.036). For Cooked Vegetables, males recorded 86.3%, compared to 95.8% for females (*p* = 0.00024).

**Figure 4:**
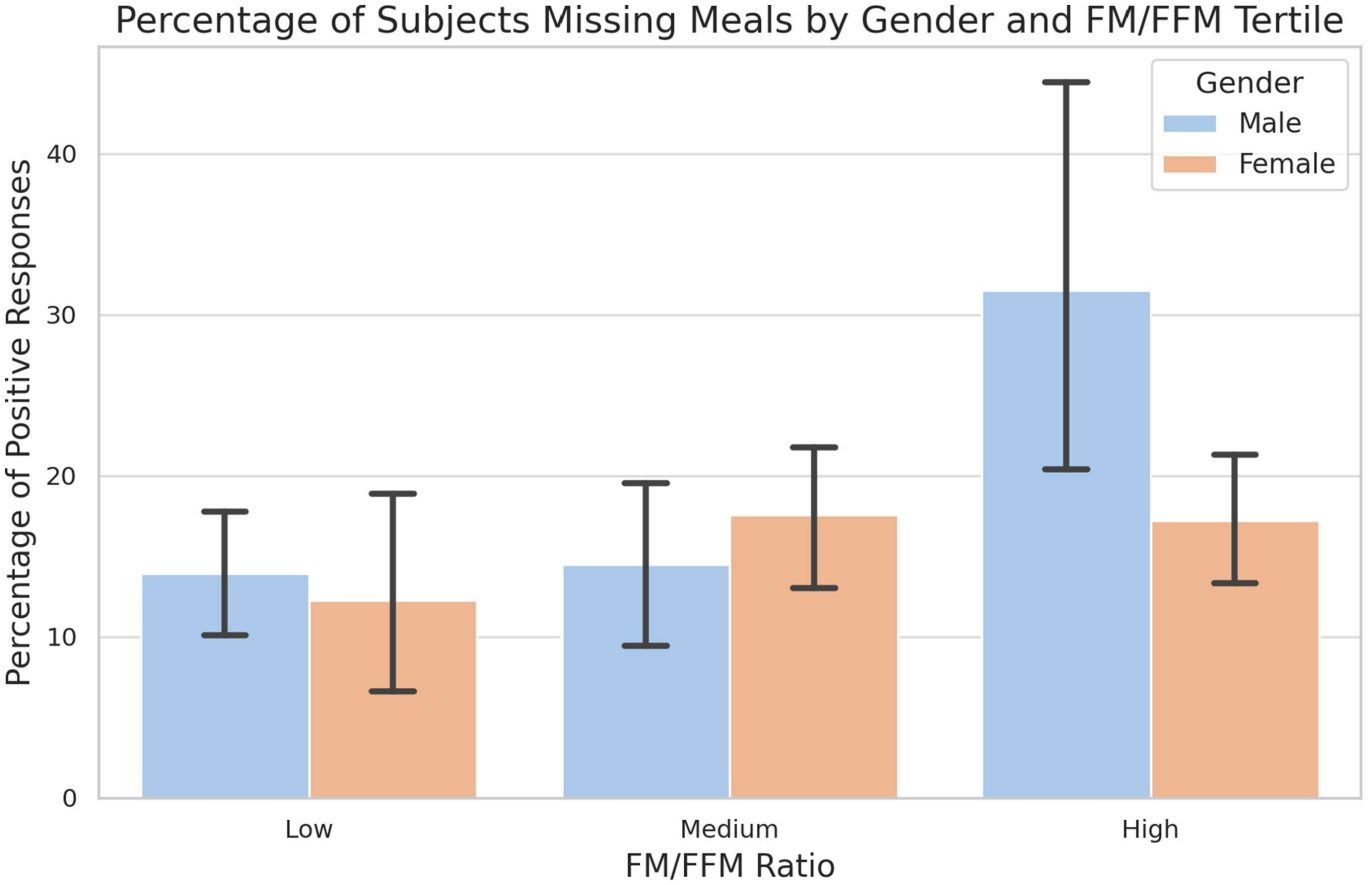
Percentage of subjects missing meals by gender and tertile of FM/FFM ratio. The percentage of male and female subjects who reported skipping meals, segmented into low, medium and high tertiles according to FM/FFM ratio. Error bars indicate the variability within each group. A chi-square test of independence reveals a significant difference in the highest tertile (LOW: *p*-value = 0.788; MEDIUM: *p*-value = 0.480; HIGH: *p*-value = 0.02).

Figure 4 shows a clear difference in the trend of missed meals between genders in the tertiles of the FM/FFM ratio. The increase in the percentage of females reporting missing meals is gradual across tertiles, from 12.3% in the low tertile to 17.5% in the middle tertile and decreasing slightly to 17.2% in the high tertile. The trend among males shows a marked contrast, particularly between the middle and high tertile. While the percentage of males skipping meals increases marginally from 13.9% in the low tertile to 14.5% in the medium tertile, there is a significant increase to 31.5% in the high tertile. Of note, a significant difference between males and females is observed in the highest tertile.

**Figure 5:**
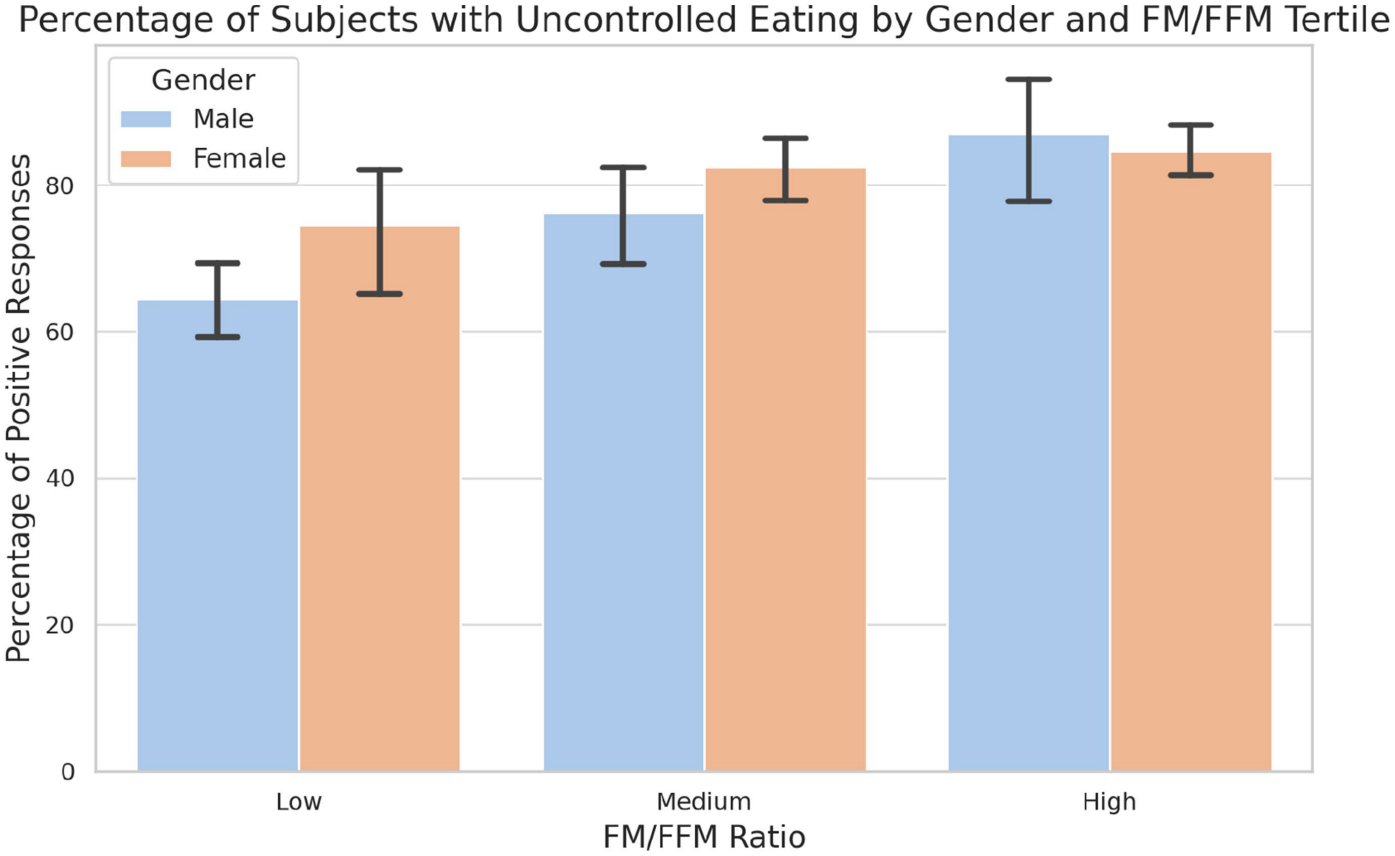
Percentage of subjects with uncontrolled eating by gender and tertile of FM/FFM ratio. The graph illustrates the prevalence of uncontrolled feeding among male and female subjects in the low, medium and high tertiles of the FM/FFM ratio. Statistical analysis reveals a significant difference in uncontrolled eating habits between tertiles and between genders in the lowest tertile (LOW: *p*-value = 0.072; MEDIUM: *p*-value = 0.137; HIGH: *p*-value = 0.792).

The analysis of the answers reveals distinct patterns of uncontrolled eating behaviour (Figure 5). These data show a gender-specific response to uncontrolled eating in relation to body composition, with a significant increase in both genders as the FM/FFM ratio increases but with a more pronounced effect in males, especially in the highest tertile. Notably, no significant differences have been observed between males and females in each tertile (LOW: *p*-value = 0.072; MEDIUM: *p*-value = 0.137; HIGH: *p*-value = 0.792).

**Figure 6:**
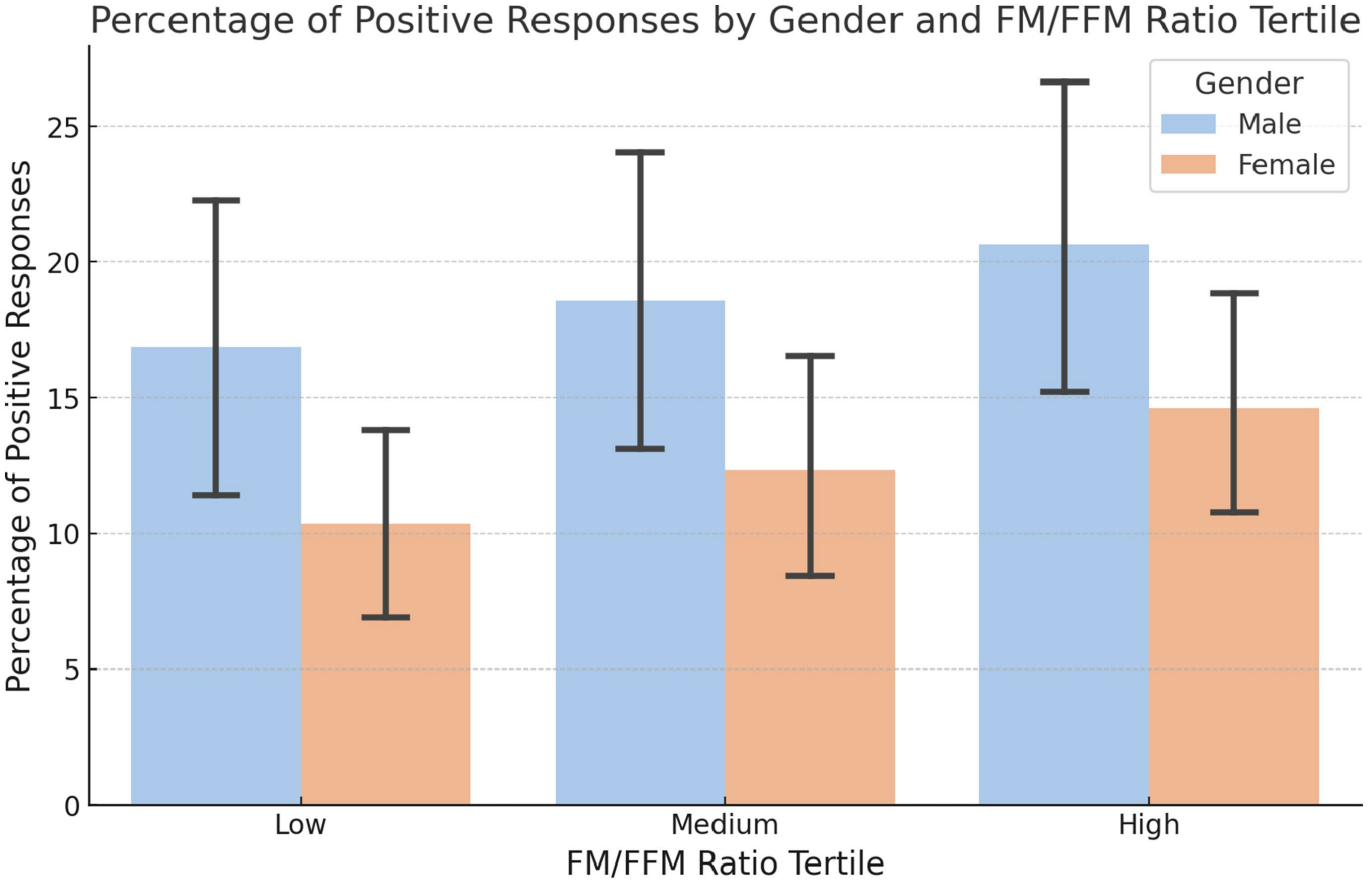
Percentage of positive responses to nighttime eating by gender and FM/FFM ratio tertile. The percentages of positive responses to the question ‘Do you wake up to eat at night?’ by gender and by tertile of the FM/FFM ratio (LOW: *p*-value = 0.162; MEDIUM: *p*-value = 0.00071; HIGH: *p*-value = 0.234).

Detailed analysis of the responses to the question on nocturnal eating habits shows interesting differences between the sexes and the various tertiles of the FM/FFM ratio. In the first tertile, 16.8% of men and 10.3% of women responded positively. In the second tertile, the percentage rises to 18.6% for men and 12.3% for women, maintaining a similar trend to the first tertile but with a slight increase for both sexes. In the third tertile, the percentages increase further to 20.7% for men and 14.6% for women (Figure 6).

**Figure 7:**
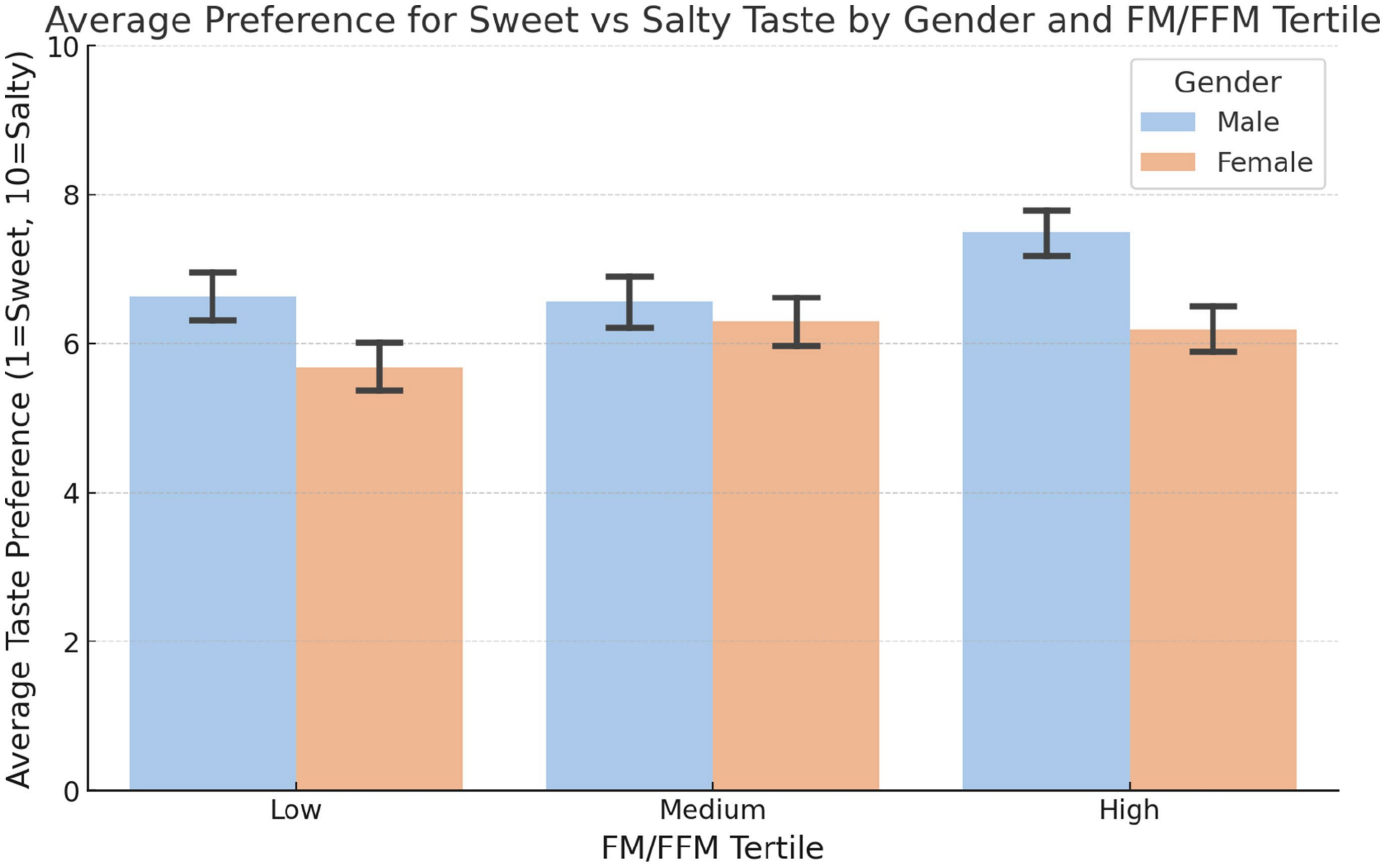
Preference for sweet vs. salty taste by gender and body composition tertiles. Mean preference for sweet over salty in the low, medium and high FM/FFM ratio tertiles for males and females. The analysis of variance (ANOVA) was utilised to determine the significance of the observed differences (LOW: *p*-value = 0.0029; MEDIUM: *p*-value = 2.58e-09; HIGH: *p*-value = 0.0063).

The results revealed significant differences in taste preference between genders and between body composition tertiles (Figure 7). Males in the high tertile showed a preference for salty tastes (mean score: 7.49), compared to those in the low (6.64) and medium (6.56) tertiles. Females showed a more subtle variation between tertiles, with scores progressively increasing from the low (5.67) to the medium (6.30) tertiles. The ANOVA test indicated that these differences were statistically significant, with *p*-values of 0.0077.

**Figure 8:**
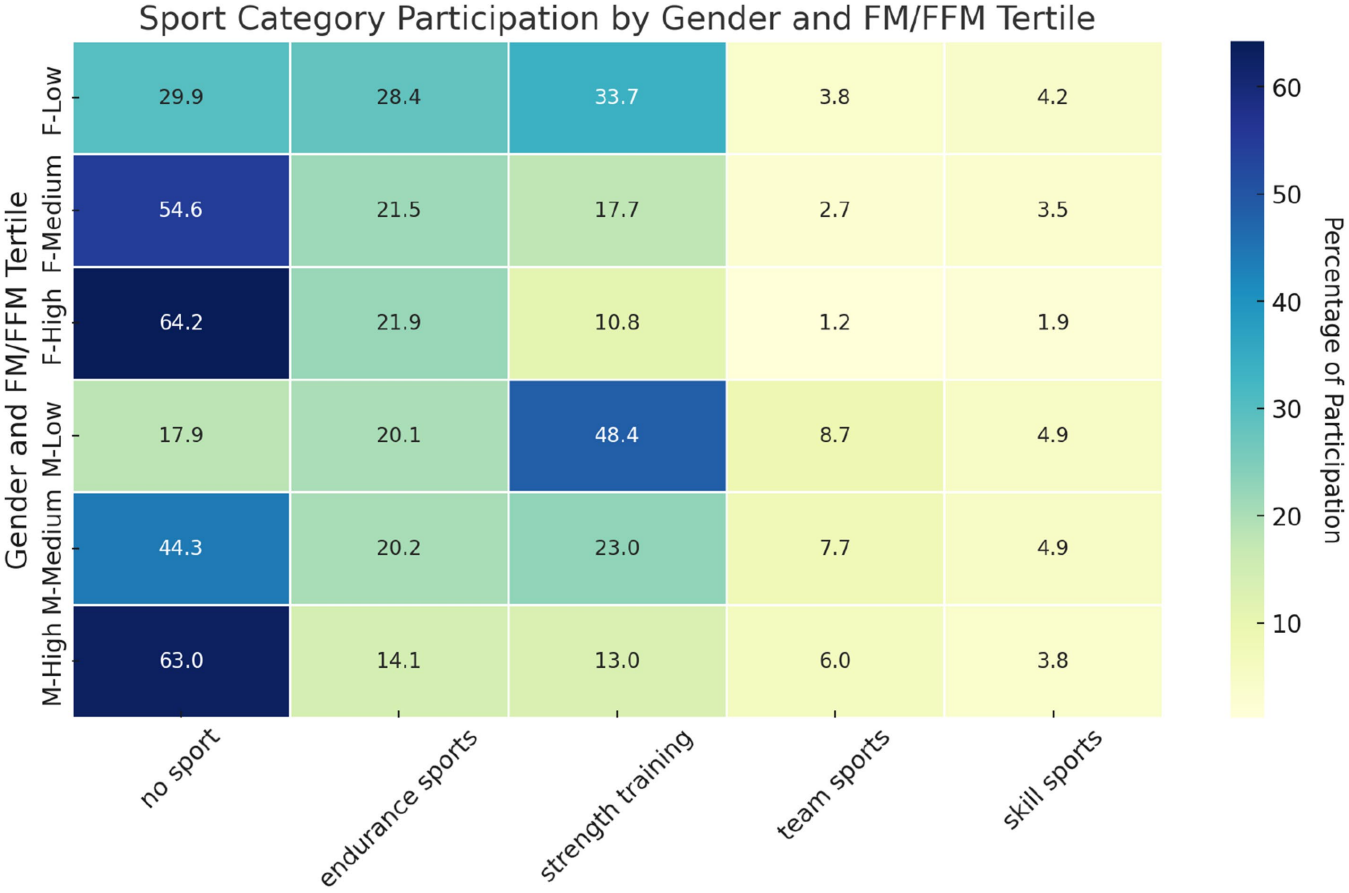
Patterns of sports participation by gender and body composition tertiles. Heatmap of sports participation rates by gender and body composition tertiles. Categories are ordered from no sport to skill sport. Chi-squared was used to test the distribution of sport participation across the categories, assessing the statistical significance of participation patterns. The overall significance was evaluated using a chi-squared test for the entire contingency table, encompassing all categories, genders, and tertiles. Statistical analysis revealed significant differences in sports participation patterns between genders and tertiles (*p*-value of 1.59 × 10^−32^).

The analysis revealed significant differences in sports participation patterns between the genders and body composition tertiles, based on the fat/lean body mass ratio (Figure 8). Sports activities have been classified into four groups: Endurance Sports, Skill Sports, Strength Training, and Team Sports (Supplementary Table S5). In men, there was a strong inclination toward Strength Training in the low tertile, with 48.4% of men choosing this category, significantly higher than in the other tertiles. Participation in No Sport increased considerably in the high tertile, reaching 63.0%. For women, the percentage of No Sport increases from 29.9% in the low tertile to 64.2% in the high tertile, showing a direct correlation between a higher FM/FFM ratio and lower sports participation. Participation in Strength Training decreases significantly from 33.7% in the low tertile to 10.8% in the high tertile. The *p*-values confirm the statistical significance of the differences observed in each combination of gender and tertile of FM/FFM, underlining the significant influence of body composition on the choice of physical activity, with values of 5.44e-28 for men in the low tertile, 3.02e-23 in the medium tertile, 2.22e-58 in the high tertile, and for women 1.49e-29 in the low tertile, 2.45e-61 in the medium, and 1.30e-94 in the high tertile.

The answers to the test questions did not reach statistical significance in the male–female comparison. They are shown in the Supplementary Figures S2–S4.

## 4 Discussion

Understanding the importance of gender differences in nutritional behaviours and body composition is becoming increasingly recognised. Sexual dimorphism of adiposity between men and women seems to be a critical determinant of food preferences. Indeed, sex hormones influence fat mass accumulation and distribution, thus leading to different food preferences, depending on the expression of estrogen/progesterone or androgen receptors in fat depots. In addition, eating habits are consistently linked to emotional or stress-related eating.

Our study presents several novel contributions to the existing literature on gender differences in body composition, dietary patterns and physical activity. First, unlike previous studies focusing on single aspects, our research integrates this information with FM-to-FFM ratios to provide a comprehensive overview of how they affect dietary preferences or physical activity levels between genders. This multifaceted approach allows for a more holistic understanding of gender-specific health behaviours and their implications. Secondly, the methodological rigour, extensively described in Methods section, ensures that our results on the relationships between FM, FFM and health behaviour are robust and reliable. Third, our study highlights the significant role of meal timing and hunger perception across different tertiles of FM-to-FFM, suggesting how circadian rhythms and hormonal differences influence gender-specific eating behaviour. This aspect has been little explored in previous research and provides new insights for the design of gender-specific interventions to improve eating habits and reduce the risk of obesity. Furthermore, we observed distinct patterns of uncontrolled and nocturnal eating in different tertiles of FM-to-FFM, particularly highlighting the pronounced differences in males with higher FM-to-FFM ratios. This finding emphasises the importance of considering body composition when developing strategies for managing eating disorders and promoting healthy eating behaviour. Finally, our analysis of sport participation patterns by gender and body composition adds a new dimension to our understanding of physical activity preferences. This information may be crucial for designing gender-sensitive physical activity programmes that address the specific needs and preferences of different body composition groups. The complex interplay between body composition, eating habits, and physical activity represents an area of growing interest in the field of nutrition and public health (18–21). Through the analysis of a significant sample of individuals, this study aimed to explore how these variables interact differentially between genders, providing a detailed perspective on how body composition may influence dietary preferences and engagement in physical activity.

A notable aspect emerging from the discussion of our results concerns gender differences in the perception of hunger during the day. The tendency of males to report a greater sense of hunger in the late afternoon and before dinner, especially in the overweight and obesity categories compared with normal weight group, highlights the complex influence of circadian rhythms and body composition on hunger perceptions and eating behaviour. Current literature, as pointed out by Davis et al. (22), emphasises that appetite-regulating hormones, such as ghrelin and leptin, exhibit circadian rhythms in their synthesis and secretion, with ghrelin stimulating appetite and leptin regulating satiety at central nervous system level. In particular, these hormones follow circadian rhythms that align with daily fluctuations in hunger, with ghrelin peaks occurring in the late afternoon and evening, corresponding to the times when males report increased hunger. Of note, obesity is associated with dysfunction in the circadian rhythms of appetite-regulating hormones, including delayed secretion of leptin from adipose depots, which may contribute to altered patterns of food intake (23). These hormonal dysfunctions, together with the leptin resistance often present in obese individuals, further complicate appetite regulation and may lead to increased food intake during periods when ghrelin levels are high.

Sex differences concerning the molecular mechanisms involved in appetite control have been recently confirmed in preclinical studies with Lipocalin 2 (Lcn2) knockout mice. This study suggests that the absence of circulatory protein Lcn2 stimulates appetite in a sex-specific manner, through higher adiponectin levels and increased AMPK phosphorylation in hypothalamus in male mice, or through reduced estradiol levels and inhibitory effects on the hypothalamic pathway ERα/PI3K/AKT in female mice (24). Meal timing plays a crucial role in influencing appetite/hunger hormonal responses, with meal timing that can help moderate satiety responses and regulate food intake. Research indicates that manipulation of meal timing, in combination with sleep timing, can have significant effects on levels of hormones such as ghrelin and glucagon-like peptide, thereby influencing satiety and caloric intake (22). Gender differences in dietary intake and appetite control (23), reveal that females are more easily satiated than males, attributable in part to gender-specific hormonal and neuronal activation. Furthermore, the differential body composition between males and females, with females tending to have a higher body fat percentage and thus higher leptin levels, plays a role in appetite regulation and energy intake. Of note, intestinal microbiota modifications have also been proposed to affect food intake in obese mice, suggesting a different contribution of the gut microbiota, associated with differences in body composition, in controlling energy balance (25, 26). In addition, different compositions in gut microbiota are observed in males and females (27) and may play a role in gender differences in the regulation of appetite. In addition, an association between food intake, meal timing and sleep disturbances has been suggested (28). Accordingly, sleep quality emerges as a key element of overall well-being, which is greatly influenced by body composition (29). Our findings indicate that sleep disturbances are more common in both genders with a high FM-to-FFM ratio, suggesting that interventions targeting body composition could improve sleep quality. Even though nocturnal eating habits are predominant in males across different tertiles of FM-to-FFM, there is the highest prevalence of night eating syndrome in the medium and high tertiles of FM/FFM of males, emphasising the need to consider gender differences in interventions designed to improve sleep quality and manage night eating (30–32). This link between sleep quality, body composition and night eating reflects the results of previous studies that have shown a correlation between fat mass index and insufficient sleep, highlighting how men and women react differently to this phenomenon (33). Furthermore, the quality of sleep prior to weight loss interventions has been associated with the success of such interventions and the maintenance of the weight lost thereafter (34). Notably, sleep alterations have also been associated with increased risk of metabolic and cardiovascular disorders (35). Studies have shown that sleep may be a modifiable factor that should be considered in weight loss programmes, potentially facilitating intervention success, with greater reduction in fat mass accumulation and improved cardiometabolic risk (36). These findings emphasise the complexity of the interactions between sleep, nutrition and body composition, underlining the importance of a holistic approach that takes these aspects into account to promote well-being and effectively manage obesity and related eating disorders (37).

We recently demonstrated the existence of differences between male and female dietary patterns in terms of food choice and eating habits (16). In this study, our findings underline the existence of significant differences in food preferences between genders, specifically influenced by body composition which proves to have a crucial role in affecting nutritional behaviours and preferences in sport activities. Particularly interesting are the gender differences in terms of preferences for animal source protein in the low (Processed Meat and Eggs) and high tertile (processed meat and red meat), where males show a greater preference than females. This data may reflect a greater emphasis on building and maintaining muscle mass, in line with cultural and social perceptions regarding masculinity and body composition (38). On the other hand, the higher preference for healthier foods like vegetable drinks and cooked vegetables among females may be influenced by increased health awareness and weight control interests, often emphasised in societal narratives targeting women (39).

The tendency of men to skip meals more frequently with an increased FM/FFM ratio could reflect a correlation between body composition and dietary decisions, influenced by considerations such as body image, personal satisfaction or metabolic factors. This observation is reflected in the study by Sun et al. (40) which shows that skipping meals is associated with an increased risk of all-cause mortality and cardiovascular disease in US adults over 40 years of age (40). In addition, research by House et al. (41) on overweight adolescents from ethnic minorities revealed that skipping meals is linked to an increase in visceral adipose tissue and triglycerides, indicators of a harmful metabolic profile. These studies show that skipping meals, although associated with a reduced daily energy intake, leads to an increase in caloric intake per eating occasion and leads to a worsening of metabolic parameters and body composition.

The subjects in our study reported episodes of loss of eating control, and the data show that the prevalence increases with higher FM/FFM ratios, especially among males. This data confirms the results obtained by Striegel-Moore and colleagues (42) investigating gender differences in the prevalence of eating disorder symptoms, including body image alterations (body control or avoidance), binge eating and inappropriate compensatory behaviour. Of the 3,714 women and 1,808 men surveyed, men revealed a higher tendency to overeating, whereas women displayed a greater likelihood of loss of control episodes during eating. These results suggest that a substantial minority of men report symptoms of eating disorders, highlighting the importance of including men in studies on eating disorders. Interestingly, a recent study found gender similarities in terms of risk factors for eating disorders development (43). On the other hand, our findings that men with higher FM/FFM ratios report a marked tendency toward lack of uncontrolled eating indicate that eating disorders need to be closely monitored and represent a health risk factor also in men.

A recent systematic review assessed whether a possible association exists between obesity and taste alterations, revealing that obesity leads to a sensitivity reduction in terms of taste (44). In our study, we found that men have a greater preference for salty taste, particularly in individuals with a higher FM-to-FFM ratio. This could reflect physiological differences or different dietary needs associated with different body compositions. Of note, females also showed variation, but the differences were less pronounced. Hormonal changes in females have recently been shown to influence taste sensitivity to some extent, nevertheless the general patterns are still unclear (45).

This study also highlights the intricate relationship between body composition and sports participation preferences. The significant disparities observed between the different FM/FFM tertiles and between genders point out the role of fitness and body composition in influencing sport activity choices. In accordance with the observations obtained by Jodkowska et al. (46), the higher prevalence of ‘no sport’ in the highest tertiles suggests a potential barrier to sport participation for individuals with higher fat mass, who need targeted interventions to be oriented toward suitable physical activity among these groups. Furthermore, the observed gender-specific preferences, in particular the male predilection for strength training in the lowest tertiles, reflect the different motivations and barriers faced by different genders in sports participation (47). These findings call for a more nuanced approach in the promotion of sport and physical activity that takes into account the complex interplay between body composition, gender and individual preferences.

Despite the significant results obtained, this study has some limitations. Firstly, the cross-sectional nature of the research does not allow causal relationships to be established between body composition, eating habits and physical activity. Secondly, self-reported data, particularly those related to eating habits and physical activity, could be subject to memory or social desirability bias. Third, although the study includes a large sample of participants, national or international representativeness may not be guaranteed, limiting the generalisability of the results. Another limitation of our study is the reliance on self-reported data, which may be subject to memory and social desirability bias. Finally, the measurement of body composition by bioimpedance analysis, although common and practical, does not reach the accuracy of other more sophisticated techniques such as DEXA. Future research could benefit from the integration of objective measures of dietary intake and physical activity, such as wearable devices or direct observations. Furthermore, longitudinal studies would help to establish causal relationships between body composition, dietary habits and physical activity.

## 5 Conclusion

The broader implication of our findings is the need for personalised approaches in the development of dietary guidelines and nutritional interventions. The significant differences in dietary preferences between men and women, influenced by body composition, highlight the limitation of one-size-fits-all approaches and suggest the value of more tailored recommendations taking into account combined contribution of gender and body composition. Future research should further explore the mechanisms underlying these preference differences and different dietary behaviours, including psychological, social and cultural factors, in addition to individual perceptions on health and well-being. In particular, our results highlight the need for gender-specific dietary guidelines that take body composition into account. In accordance with this approach, nutritional interventions targeting men with higher FM/FFM ratios could focus on reducing processed meat consumption and encouraging strength training, while strategies for women could further reinforce the tendency to consume vegetable foods also promoting endurance sports. A more in-depth investigation could provide crucial insights into the development of more effective and accepted nutritional strategies promoting healthy eating habits among different populations. While the results revealed some clear trends and significant differences between men and women, it is crucial to consider these data in light of methodological limitations and the need for further research to further deepen our understanding of these phenomena.

## Data availability statement

The raw data supporting the conclusions of this article will be made available by the authors, without undue reservation.

## Ethics statement

The studies involving humans were approved by IRCCS San Raffaele Ethics Committee (registration number RP 23/13). The studies were conducted in accordance with the local legislation and institutional requirements. The participants provided their written informed consent to participate in this study.

## Author contributions

ML: Supervision, Writing – original draft, Writing – review & editing. AF: Writing – original draft. AA: Writing – original draft. EC: Visualization, Writing – original draft. SG: Visualization, Writing – review & editing. RS: Writing – review & editing. EP: Supervision, Writing – review & editing. MC: Supervision, Writing – review & editing. AB: Supervision, Writing – review & editing.
