# Supplementary material for: Gender differences in body composition, dietary patterns, and physical activity: insights from a cross-sectional study
Source: Front Nutr. 2024 Jul 11;11:1414217. doi: 10.3389/fnut.2024.1414217 (PMC11271261; doi:10.3389/fnut.2024.1414217)
Supplement: Supplementary file 1 [file Supplementary_Material.docx]

Our analysis revealed significant differences in food preferences between genders in the various BMI categories (Figure 3). For the normal-weight group (BMI 18.5 - 24.9), cow's milk is more consumed by men with a difference of 12.6% (p = 0.0293), while dark chocolate at least 70% is preferred by women with a difference of 15.6% (p = 0.0319). Whole-grain foods are consumed more by women with a difference of 7.5% (p = 0.0271). In the overweight category (BMI 25.0 - 29.9), cooked vegetables are consumed more by women with a difference of 5.7% (p = 0.0471). For the obese class I group (BMI 30 - 34.9), raw vegetables and vegetable drinks such as soya milk are more consumed by women with differences of 9.7% (p = 0.0491) and 11.4% (p = 0.0244), respectively. Finally, for the obese class II group (BMI >35), cooked vegetables are still more consumed by women with a difference of 10.7% (p = 0.0401).

Supplementary Figure 1 - Significant differences between men and women in preferences for certain foods stratified by BMI ranges


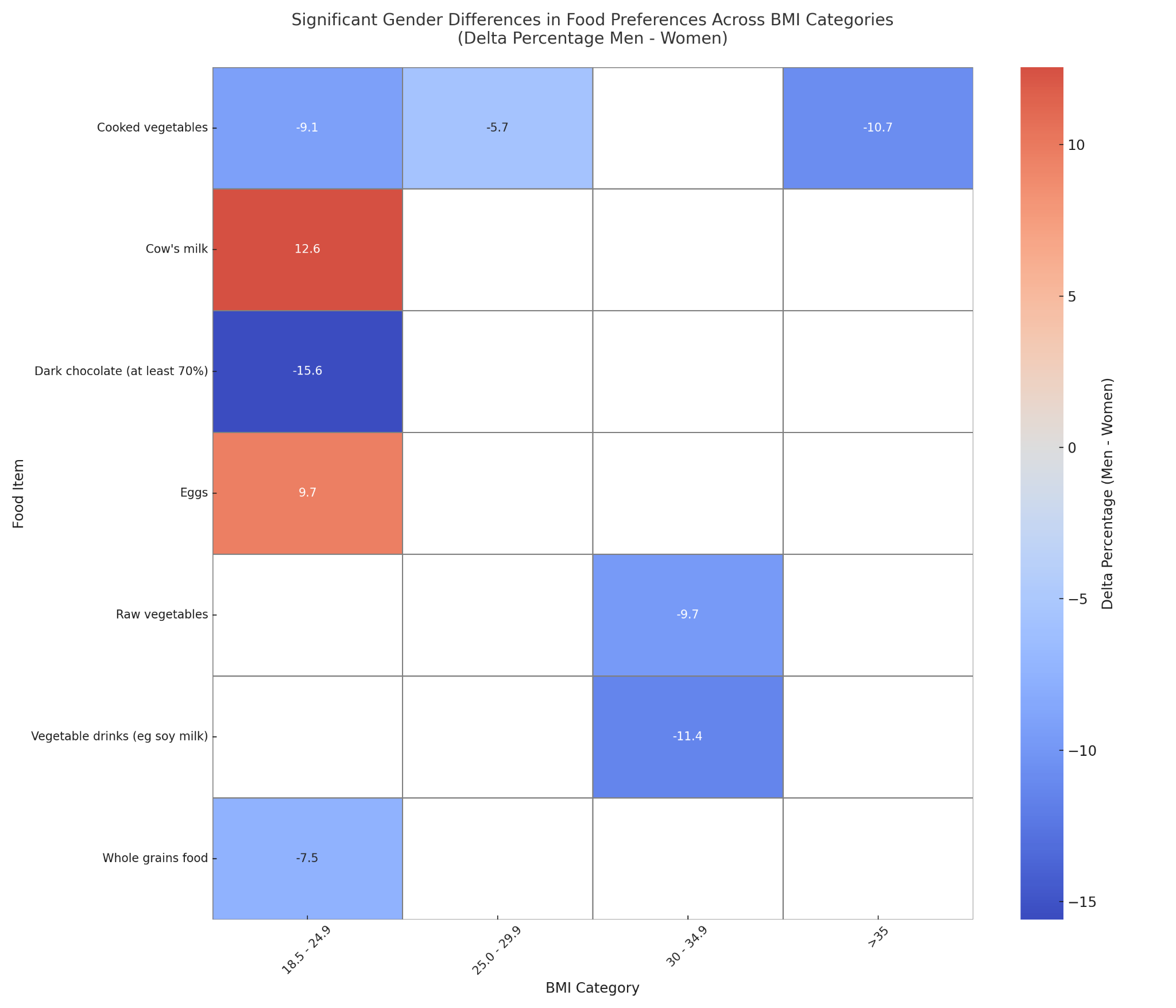


Caption Figure 1s - The heatmap shows significant differences in food preferences between men and women in different BMI categories. Statistical significance was determined using the Chi-square test. No significant differences were found in the “High” FM kg/FFM category. "Cooked vegetables" (p = 0.0047, 0.0471, 0.0401 for BMI categories "18.5 - 24.9", "25.0 - 29.9" and ">35", respectively), "cow's milk" (p = 0.0293 for "18.5 - 24. 9'), 'Dark chocolate (at least 70%)' (p = 0.0319 for '18.5 - 24.9'), 'Eggs' (p = 0.0488 for '18.5 - 24.9'), 'Raw vegetables' (p = 0.0491 for '30 - 34.9'). 0491 for '30 - 34.9'), 'Vegetable drinks (e.g. soya milk)' (p = 0.0244 for '30 - 34.9') and 'Whole Grain foods' (p = 0.0271 for '18.5 - 24.9')'.

Most of the sampled subjects claim to eat in a distracted manner, i.e. watching TV or consulting their smartphones (figure 2s). The results showed a significant difference between men and women but little difference between the tertiles of the FM/FFM ratio. Among women, in the lowest tertile, 66.0% reported eating distractedly. In the high tertile, the percentage increases to 68.2%. For males in the low tertile, 60.7% reported distracted eating. This percentage increases to 62.9% in the middle tertile. In the high tertile, the percentage rises further to 64.8%.

Supplementary Figure 2 - Percentage of Subjects Eating Distracted by Gender and Tertile of FM/FFM Ratio


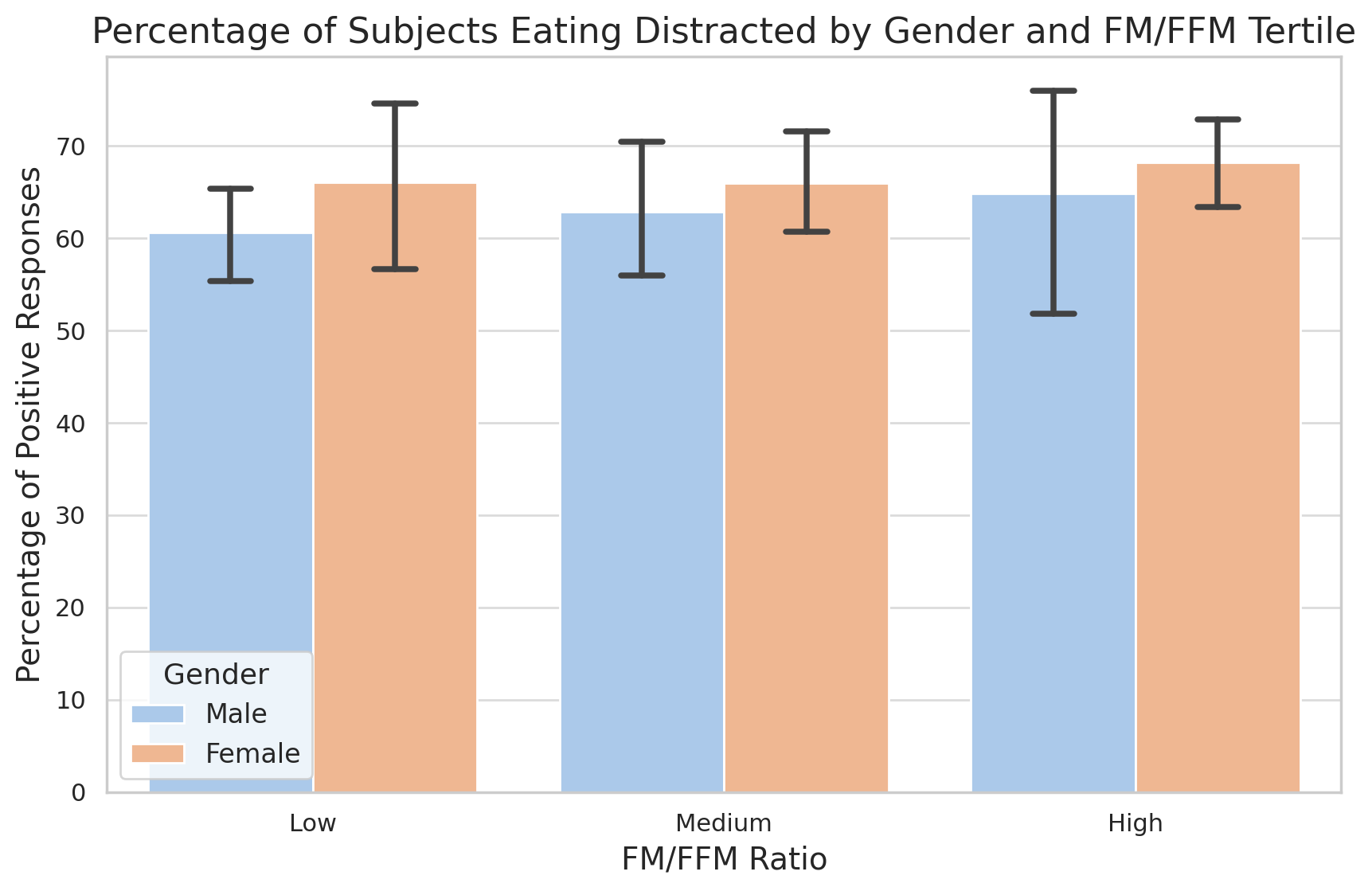


Figure 2s caption - The figure displays the percentage of subjects, broken down by gender, who eat distractedly in the low, medium and high FM/FFM tertiles. (LOW: p-value = 0.38; MEDIUM: p-value = 0.58; HIGH: p-value = 0.73)

The data show a variation in snacking behaviour between both sexes in the different body composition tertiles (figure 3s). Females show an upturn in snacking frequency from the middle (55.4%) to the high tertile (61.5%), while males show a more consistent pattern, with a slight increase in snacking frequency from the low (54.7%) to the middle tertile (58.5%).

Supplementary Figure 3 - Percentage of Subjects Snacking Between Meals by Gender and FM/FFM Tertile


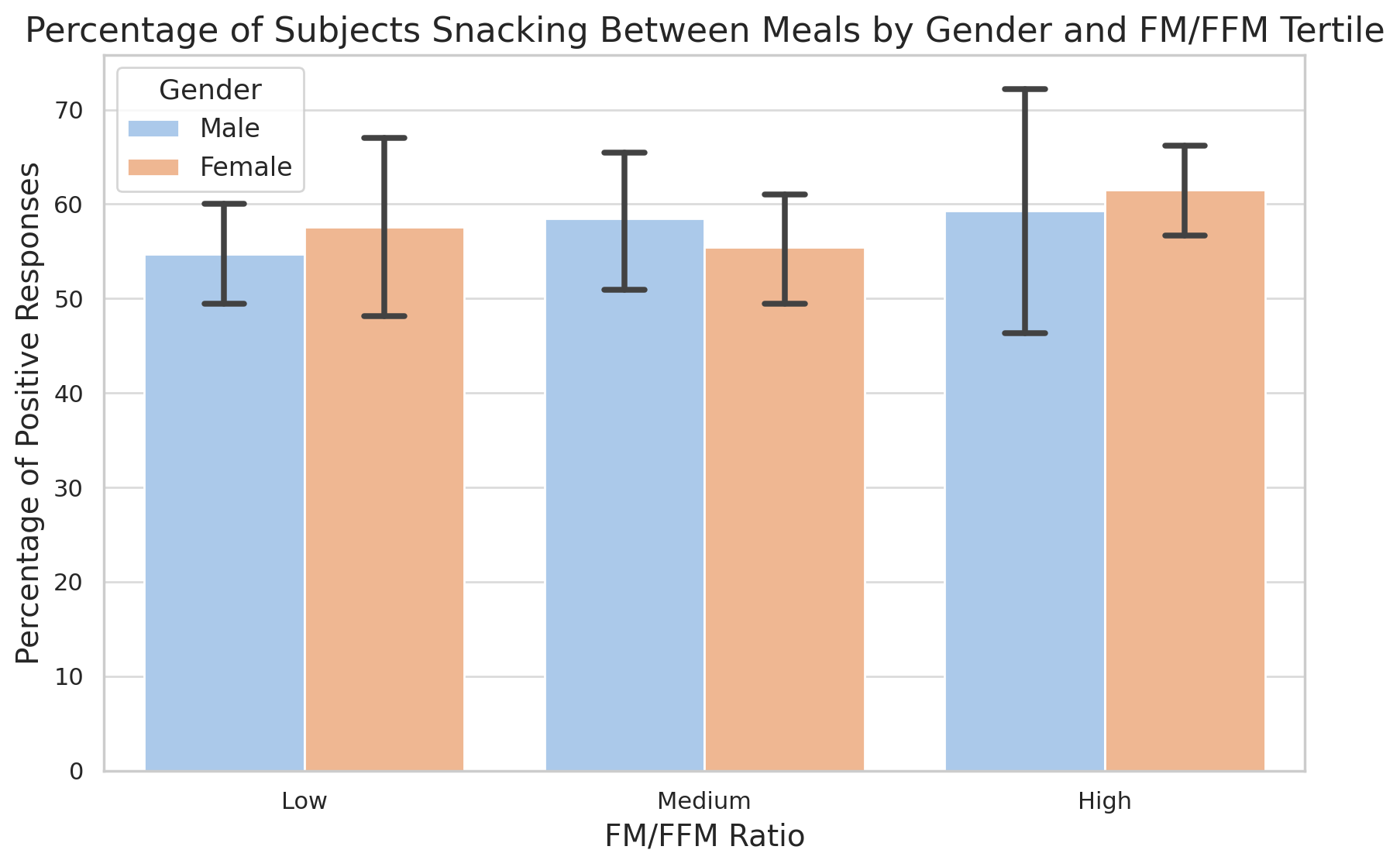


Caption figure 3s - The figure shows the percentage of male and female subjects snacking between meals, broken down by low, medium and high tertiles of the FM/FFM ratio. A chi-square test for independence reveals gender differences in snacking behaviour between tertiles (LOW: p-value = 0.395; MEDIUM: p-value = 0.000845; HIGH: p-value = 0.403)

Data analysis showed that a higher proportion of males than females report sleeping poorly in the high tertile of the FM/FFM ratio, with a less marked difference in the low and medium tertiles (Figure 4s). Negative response rates increased for both sexes as the tertile of the FM/FFM ratio increased, suggesting a possible link between higher body composition of fat mass relative to lean mass and sleep quality.

Supplementary Figure 4 - Distribution of Sleep Disorder by Gender and Tertile of FM/FFM Ratio

Caption figure 4s - The percentage of subjects who answered negatively to the question whether they think they sleep well, divided by gender and tertile of the FM/FFM ratio. Differences were tested using the chi-square test (p-value = 0.0263).


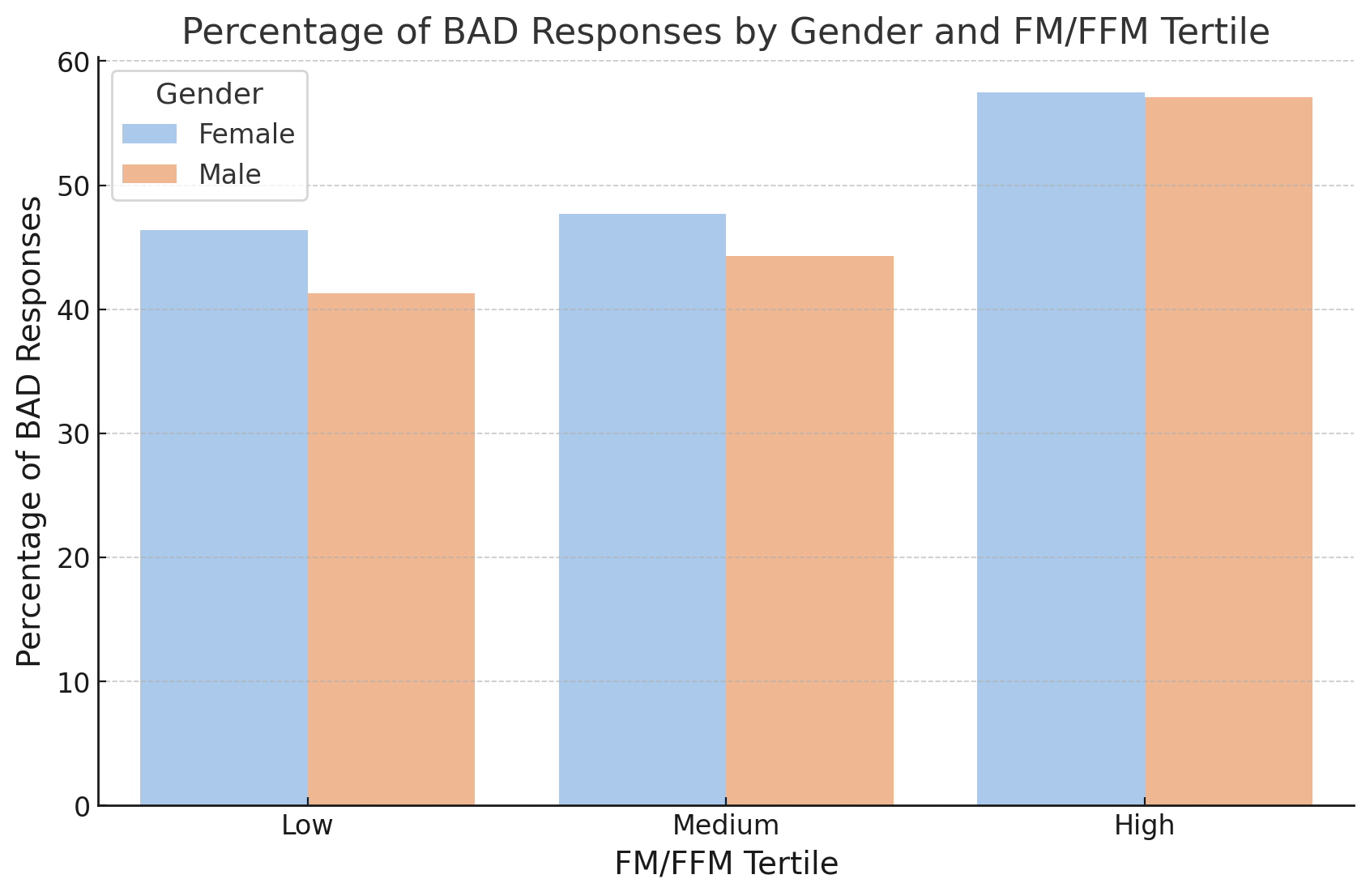


Caption figure 4s - The figure shows the percentage of male and female subjects reporting a por sleep quality, broken down by low, medium and high tertiles of the FM/FFM ratio. A chi-square test for independence reveals gender differences between tertiles (LOW: p-value = 0.337; MEDIUM: p-value = 0.538; HIGH: p-value = 0.973)

Table 5S - Classification of Sports Activities by Category

| **Endurance Sports** | **Skill Sports** | **Strength Training** | **Team Sports** |
| --- | --- | --- | --- |
| Acquagym | Boxing | Calisthenics | Acrobatic Dance |
| Cycling | Capoeira | Functional Bodybuilding | Basketball |
| Elliptical Bike | Golf | Home Work Out | Handball |
| Hydrobike | Gymnastics | Pilates | Hockey |
| Rowing | Horse Riding | Powerlifting | Rugby |
| Running | Martial arts | Pump | Soccer |
| Spinning | Padel | Weightlifting | Volleyball |
| Step | Ping Pong |  | Waterpolo |
| Total Body | Pole Dance |  |  |
| Treadmill | Skating |  |  |
| Trekking | Tai Qui |  |  |
| Walking | Tennis |  |  |
|  | Yoga |  |  |

Table 5S: Classification of sports activities into groups: Endurance Sports, Skill Sports, Strength Training, and Team Sports.
